# Clinical and genetic characterization of *DNM1l*-related disorders: insights into genotype–phenotype correlations

**DOI:** 10.3389/fped.2025.1672700

**Published:** 2025-10-30

**Authors:** Xu Manting, Li Hua, Huang Sheng, Han Xiaodi, Sun Dan, Fang Fang

**Affiliations:** ^1^ Department of Neurology, Beijing Children’s Hospital, Capital Medical University, National Center for Children's Health, Beijing, China; ^2^ Department of Neurology, Tongji Medical College, Wuhan Children’s Hospital, Huazhong University of Science & Technology, Wuhan, China

**Keywords:** *DNM1l*, genotype-phenotype correlations, RHADS, protein domain, EEG biomarker

## Abstract

**Objective:**

This study aimed to characterize the clinical and genetic spectrum of dynamin 1-like gene (*DNM1l*)-associated disorders and to investigate genotype-phenotype correlations in the largest integrated cohort reported to date.

**Methods:**

Clinical and genetic data from eleven Chinese patients with *DNM1l* variants were prospectively collected between April 2020 and May 2025. In addition, a comprehensive review of all published cases was performed.

**Results:**

A total of 66 cases were analyzed, including 11 newly reported Chinese patients and 55 previously published cases. The most common clinical manifestations were developmental delay (89.4%), epilepsy (66.7%), dystonia (53.0%), ataxia (21.2%), and failure to thrive (18.2%). Abnormal neuroimaging (80.3%) and electroencephalogram (EEG) abnormalities (78.0%) were also frequent. Domain-specific analyses demonstrated that, compared with GTPase domain variants (*n* = 15), autosomal dominant middle domain variants (*n* = 43) were associated with significantly higher risks of epilepsy, status epilepticus, cerebral atrophy, and poorer survival, but lower rates of peripheral neuropathy and ataxia (all *p* < 0.05). Nine patients with middle domain variants exhibited rhythmic high-amplitude delta with superimposed (poly)spikes (RHADS) on EEG. Within the middle domain subgroup, 67.4% developed childhood-onset status epilepticus, whereas the remaining 32.6% presented with infantile encephalopathy without status epilepticus, a phenotype associated with significantly higher mortality and earlier death (both *p* < 0.05). All 21 patients (100%) with the p.Arg403Cys hotspot variant experienced status epilepticus. The most severe phenotype was observed in two siblings with biallelic truncating variants, both of whom died in the neonatal period. Further cases are required to confirm statistically associations between variant type and clinical severity.

**Conclusion:**

This study provides the largest clinical and genetic characterization of *DNM1L*associated disorders to date and establishes genotype-phenotype correlations stratified by protein domain. The identification of RHADS as a distinctive EEG signature highlights its potential utility as a biomarker for specific clinical and genetic subgroups. Validation in larger, independent cohorts is necessary.

## 1 Introduction

Mitochondrial dynamics, encompassing both fusion and fission, are critical for maintaining cellular function. DRP1 (dynamin 1-like protein), encoded by *DNM1l*, is a member of the dynamin superfamily of GTPases and mediates mitochondrial fission (1). DRP1 is ubiquitously expressed, with the highest levels in skeletal muscle, heart, kidney, and brain. The protein comprises an N-terminal GTPase domain, a middle assembly domain, a short insert, and a GTPase effector domain (GED). In animal models, *Dnm1l*-null mice die during embryogenesis, whereas conditional deletion of *Dnm1L* in the mouse brain leads to developmental abnormalities, underscoring the essential role of this gene in mammalian development (2, 3).

Pathogenic *DNM1l* variants that disrupt mitochondrial dynamics are associated with multisystem involvement, including developmental delay, dystonia, epilepsy (most commonly refractory seizures and status epilepticus consistent with epileptic encephalopathy), ataxia, optic atrophy, microcephaly, peripheral neuropathy, respiratory distress, and childhood mortality (4). The clinical course of *DNM1l*-related disorders is highly heterogeneous and may result from *de novo* heterozygous, biallelic compound heterozygous, or homozygous recessive variants (5). Evidence from limited studies suggests that DRP1 GTPase domain variants are associated with milder phenotypes compared with middle domain variants (6). However, significant knowledge gaps remain regarding the full clinical spectrum and the precise patterns of genotype-phenotype correlation.

We reported findings from 11 Chinese patients with *DNM1l*-related disorders and integrated these with all previously published cases to conduct the largest genotype-phenotype analysis to date. Electroencephalogram (EEG) evaluation identified a distinctive pattern, termed rhythmic highamplitude delta with superimposed (poly)spikes (RHADS), associated with these disorders.

## 2 Materials and methods

### 2.1 Patients

Eleven patients with confirmed *DNM1l* variants were enrolled between April 2020 and May 2025. Eight were recruited from Beijing Children's Hospital (BCH), Capital Medical University, and three from Wuhan Children's Hospital (WCH), Huazhong University of Science and Technology. The study protocol was approved by the ethics committees of both institutions [approval nos.: (2022)-E-121Y for BCH and 2021R101-E01 for WCH]. Written informed consent for participation in the research and genetic analyses was obtained from the parents or legal guardians of all patients.

Clinical data were collected, including demographic characteristics, personal and family history, age at onset, clinical manifestations, outcomes, magnetic resonance imaging (MRI) and video EEG findings, laboratory results, and genetic data. All EEGs were interpreted by qualified neurophysiologists using the following criteria for RHADS: occipital predominance; slow rhythm (<1 Hz) of high amplitude (200–1,000 μV); frequent occurrence; and superimposed polyspikes (7). Disease prognosis was assessed using the Modified Rankin Scale (8). Patient data were obtained from the FUTang Updating Medical Records Database (9).

### 2.2 Molecular analysis

Genomic DNA was extracted from peripheral blood leukocytes of patients and their parents using standard protocols (10). Trio whole-exome sequencing was performed with an average depth of over 100×, and mitochondrial genome sequencing was conducted with an average depth exceeding 40,000×. Candidate variants identified through filtered data analysis were validated by PCR amplification of target regions followed by Sanger sequencing. Variant pathogenicity was classified according to the American College of Medical Genetics and Genomics guidelines (11, 12).

### 2.3 Literature review

**Figure 1:**
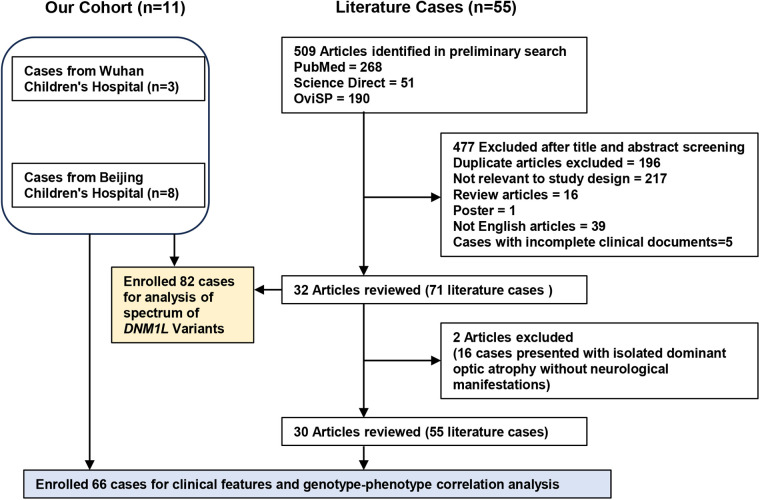
Flow chat of enrolled cases with *DNM1l*-related disorders for clinical and genetic analysis.

A literature review was conducted using the keywords “*DNM1l*”, “*DNM1l* mutation”, and “*DNM1l* variant” across three electronic databases (PubMed, ScienceDirect, and OviSP) from January 2007 to June 2025 (Figure 1). Cases with incomplete clinical documentation or duplicate reports were excluded. Clinical data, laboratory findings, and genotypic profiles were extracted.

RHADS were identified by certified EEG specialists who retrospectively reviewed the published EEG descriptions and images. To facilitate genotype-phenotype correlation analysis, patients with DNM1l-associated disorders from both our Chinese cohort and the literature were categorized into four groups according to inheritance pattern and variant location: GTPase domain/autosomal dominant (AD), middle domain/AD, GED domain/AD, and GTPase domain/autosomal recessive (AR).

### 2.4 Statistical analysis

Quantitative variables were summarized as medians with interquartile ranges. Continuous data were compared between groups using the Mann–Whitney *U*-test, whereas categorical variables were expressed as frequencies and percentages. Survival analysis was performed using the logrank test. *P*-values < 0.05 were considered statistically significant. Statistical analyses were conducted with IBM SPSS Statistics version 20 (IBM Corp., Armonk, NY, USA), and graphs were generated using GraphPad Prism version 8.

## 3 Results

### 3.1 Case series

This study included 11 Chinese patients (five females and six males) with no evidence of consanguinity. Prenatal, perinatal, and family histories were unremarkable. The median age at onset was 3.3 years (range, 1.5–5 years). Developmental delay was observed in 7 of 11 patients (63.6%). Patient 1 exhibited developmental delay and dystonia from birth and later developed peripheral neuropathy; however, epilepsy did not manifest until 4.7 years of age. All remaining 10 patients developed drug-resistant epilepsy, of whom eight progressed to status epilepticus accompanied by respiratory failure. Reported seizure types included focal seizures (*n* = 5), myoclonic seizures (*n* = 7), generalized tonic-clonic seizures (*n* = 3), and epileptic spasms (*n* = 1). During a median follow-up of 1.4 years (range, 0.4–4.7 years), two patients discontinued treatment and were lost to follow-up. The remaining nine patients (median age, 4.7 years; range, 0.7–10.5 years) were alive at the last assessment, with persistent manifestations such as cognitive impairment and motor delay. Notably, all eight patients with epilepsy continued to experience uncontrolled seizures despite intensive management with antiseizure medications and mitochondrial support. At the final evaluation, the median Modified Rankin Scale score was 3 (range, 2–5) (Supplementary Table S1).

Muscle biopsy was performed in one patient and yielded unremarkable findings. Hyperlactatemia was detected in 44.4% of cases (four of nine). During the acute disease phase, brain MRI abnormalities were observed in 6 of 10 patients (60%), including thalamic lesions (*n* = 1), cortical lesions (*n* = 3), white matter lesions (*n* = 1), and thinning of the corpus callosum (*n* = 1). Followup MRI performed 1.5–6 months after disease onset in all eight re-examined patients (100%) revealed new-onset cerebral atrophy. Video EEG abnormalities were present in all ten patients with epilepsy, including background slowing (*n* = 4), focal epileptiform discharges (*n* = 5), multifocal epileptiform discharges (*n* = 4), and generalized epileptiform discharges (*n* = 1).

**Figure 2:**
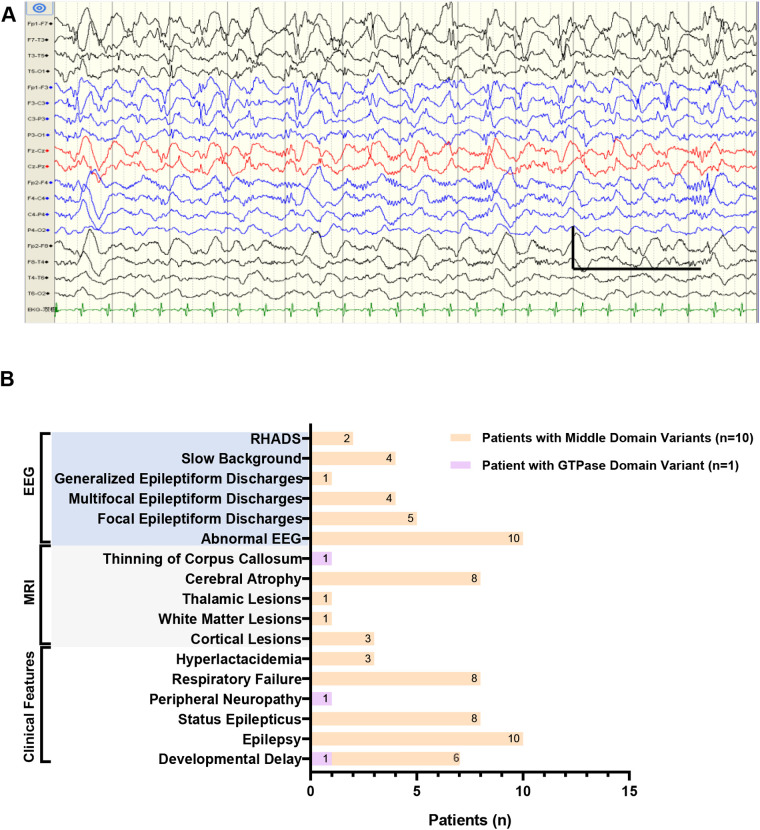
Clinical features in our Chinese cohort. **(A)** The electroencephalogram (EEG) of patient 4 in Chinese cohort showed typical RHADS in the bilateral frontal and central regions (1s,100uv,1.0 Hz,70 Hz). **(B)** Distribution of clinical features in our Chinese cohort of 11 patients harboring *DNM1l* variants in the GTPase or middle domain. RHADS: rhythmic high amplitude delta waves with superimposed (poly)spikes, EEG: electroencephalogram, MRI: magnetic resonance imaging.

Among those with focal epileptiform discharges, two patients (Patients 4 and 5) who developed status epilepticus and carried the heterozygous p.Arg403Cys variant demonstrated RHADS on EEG (Figure 2A). The predominant epileptiform abnormalities included spikes, spike-and-wave complexes, and polyspike-wave complexes. Detailed clinical information is provided in Figure 2B and Supplementary Table S1.

**Figure 3:**
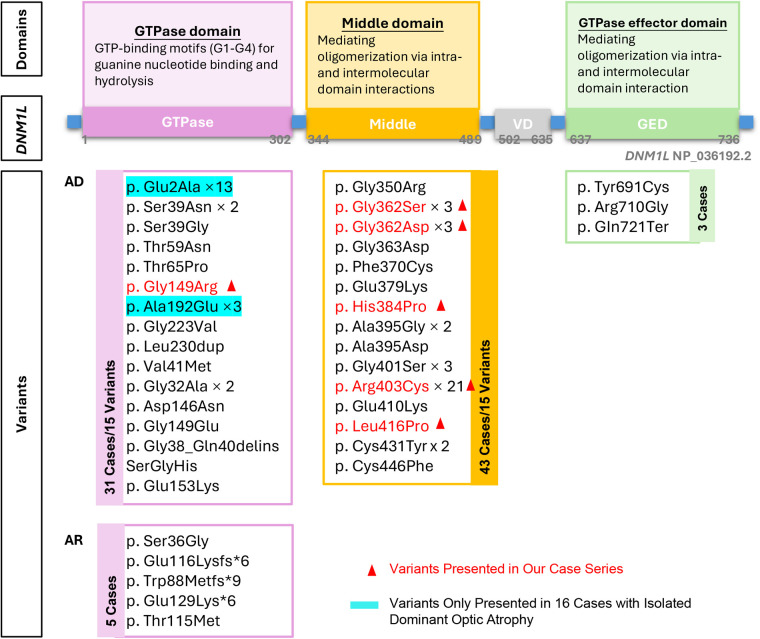
Overview of the domain structure of *DNM1l*, the identiffed variants at each domain. AD: autosomal dominant inheritance, AR: autosomal recessive inheritance.

Six distinct *de novo DNM1l* variants were identified: p.Gly149Asn, p.Gly362Ser, p.Gly362Asp, p.Arg403Cys, p.Leu416Pro, and p.His384Pro. Patient 1 carried the p.Gly149Asn variant located in the GTPase domain, whereas the remaining 10 patients harbored variants in the middle domain (p.Gly362Ser, p.Gly362Asp, p.Arg403Cys, p.Leu416Pro, and p.His384Pro). Among these, the p.Arg403Cys variant was the most frequent, accounting for 54.5% (6 of 11) of cases (Figure 3).

### 3.2 Literature review

#### 3.2.1 Clinical summary

A total of 82 patients with *DNM1l*-associated disorders have been reported across 32 articles from 2007 to June 2025, including the cases described in this study (4–6, 13–41). Of these, 16 patients presented with isolated dominant optic atrophy without neurological manifestations (13, 14). Consequently, the following analysis focuses on the remaining 66 patients of diverse ethnic and national backgrounds.

The median age at onset was 2.4 years (range, 0–14 years). The most common manifestations were developmental delay (59/66, 89.4%), epilepsy (44/66, 66.7%), dystonia (35/66, 53.0%), status epilepticus (32/66, 48.5%), ataxia (14/66, 21.2%), and failure to thrive (12/66, 18.2%). Additional features included microcephaly (*n* = 6), cardiomyopathy (*n* = 7), optic atrophy (*n* = 5), and peripheral neuropathy (*n* = 9). Follow-up data were available for 51 patients, among whom 17 deaths were reported.

Brain MRI abnormalities were reported in 80.3% (49 of 61) of cases, including cortical lesions (*n* = 10), basal ganglia or brainstem lesions (*n* = 17), white matter lesions (*n* = 8), cerebral atrophy (*n* = 29), and corpus callosum thinning or agenesis (*n* = 9). An elevated lactate peak on magnetic resonance spectroscopy was observed in 36.4% (4 of 11) of cases. EEG abnormalities were identified in 78% (32 of 41), most commonly diffuse slow-wave background activity (*n* = 21) and epileptiform discharges (*n* = 29). Re-analysis of 30 cases (13 articles with detailed EEG data along with our cohort) identified RHADS as a distinctive EEG feature in 9 patients. Hyperlactatemia was reported in 49.1% (27 of 55). Muscle biopsy, performed in 19 patients, demonstrated decreased respiratory chain enzyme activity in 6 cases and abnormal mitochondrial morphology in 5 cases.

#### 3.2.2 *DNM1l* variants

To date, 38 distinct pathogenic or likely pathogenic variants have been reported in 82 cases (4–6, 13–41). Of these, 93.9% (77 of 82) exhibited an AD inheritance pattern, encompassing 33 distinct variants: 15 in the GTPase domain, 15 in the middle domain, and 3 in the GED domain. The remaining 5 cases showed AR inheritance, all involving variants in the GTPase domain (Figure 2). The p.Arg403Cys variant was identified in 21 patients, establishing this residue as a mutational hotspot of the *DNM1l* gene across diverse ethnic backgrounds. In addition, missense variants affecting glycine residue 362 were observed in six cases (p.Gly362Ser, *n* = 3; p.Gly362Asp, *n* = 3), suggesting a secondary hotspot at this position.

#### 3.2.3 Genotype-phenotype correlation

**Figure 4:**
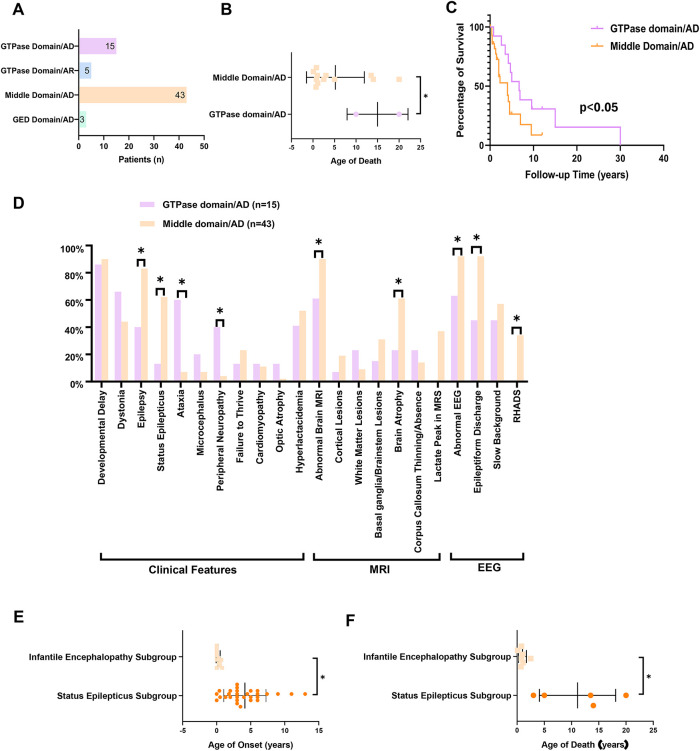
The clinical and genetic spectrum and genotype-phenotype correlation analysis of DNM1l-related disorders. **(A)** The patients’ number in GTPase domain/AD group (*n* = 15), middle domain/AD group (*n* = 43), GED domain/AD group (*n* = 3), and GTPase domain/AR group (*n* = 5). **(B)** The age of death in GTPase vs. middle domain/AD group patients. **(C)** Survival analysis between GTPase and middle domain/AD group. **(D)** Distribution of the clinical features of patients in GTPase domain/AD group and middle domain/AD group. **(E)** The age of death in status epilepticus subgroup vs. infantile encephalopathy subgroup. **(F)** The age of onset in status epilepticus subgroup vs. infantile encephalopathy subgroup. * *P* < 0.05. AD: autosomal dominant inheritance, AR: autosomal recessive inheritance, RHADS: rhythmic high amplitude delta waves with superimposed (poly)spikes, EEG: electroencephalogram, MRI: magnetic resonance imagin.

A total of 66 cases (including our cohort and literature cases) were stratified into four groups based on inheritance pattern and variant location: GTPase domain/AD group (*n* = 15), middle domain/AD group (*n* = 43), GED domain/AD group (*n* = 3), and GTPase domain/AR group (*n* = 5), to enable domain-specific phenotype analysis (Figure 4A).

##### 3.2.3.1 Comparison of middle and GTPase domain/AD variants

The median age of onset was 2.5 years (range, 0–14 years) in the GTPase domain/AD group and 2.0 years (range, 0–13 years) in the middle domain/AD group (*p* = 0.39). The middle domain/AD group exhibited significantly higher rates of epilepsy (83.7% vs. 40%), status epilepticus (67.4% vs. 13.4%), and cerebral atrophy (61.0% vs. 45.5%) compared with the GTPase domain/AD group (all *p* < 0.05; Table 1). Conversely, the GTPase domain/AD group showed higher frequencies of peripheral neuropathy (40% vs. 4.7%) and ataxia (60% vs. 7%) (both *p* < 0.05; Table 1). Other clinical features—including developmental delay, dystonia, failure to thrive, microcephaly, cardiomyopathy, and optic atrophy—occurred at comparable frequencies between the two groups. EEG analysis revealed a significantly higher burden of epileptiform discharges in the middle domain/AD group (*p* < 0.05). Notably, RHADS was observed exclusively in nine middle domain/AD cases with status epilepticus, five of whom carried the p.Arg403Cys variant.

**Table 1:** Summary of key clinical features in patients with DNM1l variants in middle and GTPase domain/AD groups.

Clinical data %(n/n)	GTPase domain/AD (*n* = 15)	Middle domain/AD (*n* = 43)
Median age of onset (years)	2.5 (range 0–14)	2 (range 0–13)
Died at the last follow-up	13.3% (2/13)	39.5% (12/30)
Development delay	86.7% (13/15）	90.7% (39/43)
Dystonia	66.7% (10/15)	44.2% (19/43)
Epilepsy	40.0% (6/15)	83.7% (36/43)*
Status epilepticus	13.3% (2/15)	62.8% (27/43)*
Ataxia	60.0% (9/15)	7.0% (3/43)*
Microcephalus	20% (3/15)	7.0% (3/43)
Peripheral neuropathy	40% (6/15)	4.7% (2/43)*
Failure to thrive	13.3% (2/15)	23.3% (10/43)
Cardiomyopathy	13.3% (2/15)	11.6% (5/43)
Optic atrophy	13.3% (2/15)	2.3% (1/43)
Abnormal brain MRI	61.5% (8/13)	90.2% (37/41)*
Cortical lesions	7.7% (1/13)	19.5% (8/41)
White matter lesions	23.1% (3/13)	9.8% (4/41)
Basal ganglia/brainstem lesions	15.4% (2/13)	31.7% (13/41)
Cerebral atrophy	23.1% (3/13)	61.0% (25/41)*
Corpus callosum thinning/absence	23.1% (3/13)	14.6% (6/41)
Lactate peak in MRS	-	37.5% (3/8)
Abnormal EEG	63.6% (7/11)	92.3% (24/26)*
Epileptiform discharge	45.5% (5/11)	92.3% (24/26)*
Slow background	45.5% (5/11)	57.7% (15/26)
RHADS	0/11	34.6% (9/26)*
Hyperlactacidemia	41.7% (5/12)	52.6% (20/38)
Abnormal in muscle biopsy	33.3% (1/3)	78.6% (11/14)
Abnormal morphology of mitochondria	0/3	35.7% (5/14)
Decreased respiratory chain enzyme activity	0/3	28.6% (4/14)
Nonspecific findings	33.3% (1/3)	28.6% (4/14)

* Significantly different between middle and GTPase domain/AD group, *p* < 0.05.

Mortality differed markedly between groups. At last follow-up, two deaths (13.3%) were reported in the GTPase domain/AD group, occurring at ages 10 and 20 years, whereas the middle domain/AD group recorded 17 deaths (39.5%) with a median age at death of 1.8 years (range, 0.1–20 years). Survival analysis demonstrated a 4.2-fold higher mortality risk in the middle domain/AD group (95% CI, 1.8–9.6; log-rank *p* < 0.05) (Figures 4B–D).

##### 3.2.3.2 Phenotypic heterogeneity in middle domain/AD group

Within the middle domain/AD group, 67.4% (29 of 43) of patients developed status epilepticus. This subgroup demonstrated a median age of disease onset of 3.5 years (range, 0–13) and seizure onset of 4.25 years (range, 1–13). At last follow-up, mortality was 26.3% (5 of 19), with a median age at death of 5 years (range, 3–20). Notably, all 21 patients carrying the p.Arg403Cys variant experienced status epilepticus. The remaining 8 cases carried variants including p.Gly362Asp (*n* = 2), p.Gly362Ser (*n* = 1), p.Phe370Cys (*n* = 1), p.Gly350Arg (*n* = 1), p.His384Pro (*n* = 1), and p.Gly401Ser (*n* = 2).

Conversely, the remaining 14 patients (32.6%) without status epilepticus presented with infantile encephalopathy. This subgroup exhibited significantly earlier symptom onset (median: 0 years; range, 0–0.8; *p* < 0.05) compared with the status epilepticus subgroup. Core clinical features included developmental delay (100%), dystonia (71.4%), failure to thrive (57.1%), respiratory distress (28.6%), and seizures (21.4%). Moreover, this subgroup showed significantly higher mortality [63.6% (7 of 11); *p* < 0.05] and an earlier median age at death (0.9 years; range, 0.1–2.5; *p* < 0.05) than patients with status epilepticus (Figures 4E–F).

##### 3.2.3.3 GED domain/AD group

Only three cases within the GED domain/AD group have been reported to date. The p.Gln721Ter variant was associated with a distinctive phenotype characterized by paroxysmal hemiplegia, astigmatism, and strabismus. Conversely, the p.Arg710Gly and p.Tyr691Cys variants were linked to the core features of *DNM1l*-associated disorders, including dystonia, developmental delay, and seizures (Supplementary Table S2).

##### 3.2.3.4 GTPase domain/AR group

Five variants (Figure 3) were identified in the AR state in five patients from three families. In two families, asymptomatic parents were confirmed to be heterozygous carriers of the following variants: p.Ser36Gly, p.Glu116Lysfs6, and p.Thr115Met. Two siblings with biallelic truncating variants (p.Trp88Metfs9 and p.Glu129Lysfs6) presented with severe dystonia and respiratory distress at birth and died at 8 and 21 days, respectively—representing the most severe phenotype reported to date in *DNM1l*-associated disorders. The remaining three patients exhibited profound developmental delay and dystonia from birth but survived, with ages ranging from 3 to 16 years at last follow-up (Supplementary Table S2).

## 4 Discussion

Mitochondria are highly dynamic organelles whose structure and organization are continuously remodeled through coordinated processes of division, fusion, and transport. *DNM1l* encodes DRP1, a highly conserved GTPase that is the primary mediator of mitochondrial and peroxisomal fission. In response to cellular signals, cytosolic DRP1 translocates to the mitochondrial outer membrane, where its distinct structural domains interact with specific receptors. The GTPase domain binds outer membrane receptors, the middle domain facilitates DRP1 oligomerization, and the GED enhances GTPase activity while stabilizing the DRP1 homodimer complex. Pathogenic *DNM1l* variants disrupt DRP1 oligomerization, impair GTPase-dependent constriction, and block mitochondrial fission, ultimately driving a broad spectrum of neurological disorders (21, 23, 27). Here, we described a cohort of 11 Chinese children with *DNM1l* variants, enriching the clinical and genetic information from the Chinese population, to improve our understanding of this rare disorder. The clinical phenotypes of these patients were consistent with previously reported cases. Notably, in a previously healthy school-aged child who developed status epilepticus clinically mimicking febrile infection-related epilepsy syndrome or Rasmussen encephalitis, immediate genetic testing was warranted to identify monogenic etiologies such as *DNM1l* variants. We conducted a comprehensive analysis of 66 patients with *DNM1l* variants, combining our Chinese cohort with published cases (excluding 16 patients with isolated dominant optic atrophy) (4–6, 13, 41). The age of onset ranged from birth to 14 years, with core manifestations including developmental delay, dystonia, epilepsy, ataxia, and failure to thrive. Additional features included microcephaly, cardiomyopathy, optic atrophy, and peripheral neuropathy. Although cardiomyopathy is rare in *DNM1l*-related phenotypes, it can present as an life-threatening feature that demands clinical vigilance. At last follow-up, one-third of patients had died. Most patients exhibited abnormal neuroimaging and EEG findings, with MRI abnormalities characterized by brain lesions, cerebral atrophy, and corpus callosum thinning or absence, whereas EEG alterations predominantly included diffuse slow-wave background activity and epileptiform discharges.

Pathogenic *DNM1l* variants are predominantly missense mutations, with several recurrent variants identified in unrelated patients. Residues Arg403 and Gly362 represent mutational hotspots, whereas other variants are distributed across the gene, clustering mainly within the GTPase and middle domains. *DNM1l*-related disorders display both dominant and recessive inheritance patterns, although most pathogenic variants occur in the heterozygous state. Domain-specific phenotype analysis demonstrated clear differences between patients with heterozygous variants in the GTPase and middle domains. Middle domain variants were associated with significantly higher risks of epilepsy, status epilepticus, cerebral atrophy, and poorer clinical outcomes. Importantly, RHADS on EEG were observed exclusively in patients with middle domain variants. Conversely, carriers of GTPase domain variants exhibited comparatively stable clinical courses, indicating milder pathogenicity, with peripheral neuropathy and ataxia occurring more frequently in this group.

Given that most patients carried heterozygous middle domain variants, we further analyzed phenotypic heterogeneity within this domain. Two distinct subgroups were identified.

Approximately two-thirds developed childhood-onset status epilepticus, with a mortality rate of 26.3% and a median age at death of 5 years. Notably, all 21 patients harboring the p.Arg403Cys variant presented with status epilepticus. Conversely, the remaining one-third, who did not develop status epilepticus, uniformly manifested infantile encephalopathy and showed markedly higher mortality with earlier death.

With only three reported cases harboring GED domain variants, phenotypic analysis remains limited. Nonetheless, a distinctive phenotype characterized by paroxysmal hemiplegia, astigmatism, and strabismus has been described, suggesting a potential GED-specific manifestation.

Only five pathogenic variants with recessive inheritance have been reported, all located in the GTPase domain. The two siblings with biallelic truncating variants presented the most severe phenotype, whereas three patients with biallelic missense variants demonstrated comparatively milder courses. These findings suggest that clinical severity may be influenced by variant type, although confirmation in larger cohorts is needed.

We also described the distinctive RHADS pattern in *DNM1l*-associated disorders. This EEG signature, previously described in mitochondrial diseases such as Alpers syndrome (7), is a hallmark of mitochondrial encephalopathy. In our re-analysis of 30 cases (including 13 published reports with EEG data and our cohort), RHADS was observed in 9 patients. All affected individuals presented with refractory status epilepticus and carried heterozygous variants in the middle domain of DRP1, five of whom harbored the recurrent p.Arg403Cys variant. These findings suggest that RHADS may serve as an early diagnostic marker and a prognostic indicator of disease severity. Larger studies are needed to validate its association with clinical and genetic features.

This study has several limitations. Although combining our cohort with retrospective literature data increased the sample size, it also introduced potential bias. Variability in clinical reporting, follow-up duration, and diagnostic methods across published cases may have affected comparability. In addition, heterogeneity in subgroup sizes limits the robustness of statistical analyses and raises the risk of overinterpretation, particularly in smaller subgroups. Despite these limitations—common to studies of ultra-rare diseases—our findings provide meaningful insights.

Validation through large-scale, prospective studies remains essential.

## 5 Conclusion

Our study provides a comprehensive clinical and genetic characterization of *DNM1l*-related disorders, underscoring genotype–phenotype correlations according to protein domain involvement. Importantly, we identify the distinctive RHADS electrographic signature as a potential EEG biomarker associated with specific clinical and genetic features. Validation of these findings in larger cohorts will be essential.

## Data Availability

The raw data supporting the conclusions of this article will be made available by the authors, without undue reservation.
