# Supplementary material for: Clinical and genetic characterization of DNM1l-related disorders: insights into genotype–phenotype correlations
Source: Front Pediatr. 2025 Oct 30;13:1672700. doi: 10.3389/fped.2025.1672700 (PMC12611810; doi:10.3389/fped.2025.1672700)
Supplement: Supplementary file 1 [file Table1.docx]

Table S1. Summary of clinical and genetical features of 11 Chinese patients with *DNM1L* variants

| **ID** | | **Sex** | **Onset age** | | **Age of report** | **Main symptoms** | | **Onset age of seizures** | | **seizure types** | | **Treatment (anesthetics and ASMs)** | | | **Treatment (others)** | | |
| --- | --- | --- | --- | --- | --- | --- | --- | --- | --- | --- | --- | --- | --- | --- | --- | --- | --- |
| P1 | | M | birth | | 4y, 8mo | Global hypotonia and developmental delay since birth, hypotonia, sensory neuropathy and ataxia | | - | | - | | - | | | 1.Mitochondrial cocktail therapy | | |
| P2 | | M | 4.5y | | 5y | seizures, status epilepticus, developmental delay, dysuria needed urethral catheterization, respiratory failure | | 4.5y | | myoclonus, focal seizures | | TPM, OXC, CZP | | | 1.Mitochondrial cocktail therapy | | |
| P3 | | F | 6y | | 7y, 8mo | seizures, status epilepticus, developmental delay, respiratory failure | | 6y | | focal seizures, myoclonus | | 1.midazolam 2.LEV, CZP, LTG, VPA, TPM, LCM | | | 1.Mitochondrial cocktail therapy 2.Mechanical ventilation | | |
| P4 | | M | 6.5y | | 7y | seizures after vaccination, status epilepticus, respiratory failure | | 6.5y | | myoclonus | | 1.midazolam 2.OXC, TPM, LEV, PB, CZP, VPA, LCM 3.Ketogenic diet | | | 1.Mechanical ventilation 2.Mitochondrial cocktail therapy | | |
| P5 | | F | 3y | | 4y, 3mo | seizures, status epilepticus, developmental delay, respiratory failure | | 3y | | myoclonus, focal seizures | | 1.midazolam 2.VPA, OXC, CZP | | | 1.Mechanical ventilation 2.Mitochondrial cocktail therapy | | |
| P6 | | F | 3y, 7mo | | 4y | seizures, status epilepticus, respiratory failure | | 3y, 7mo | | myoclonus | | 1.midazolam 2.VPA, LEV, TPM, LTG, ZNS | | | 1.Mechanical ventilation;  2.Mitochondrial cocktail therapy | | |
| P7 | | F | 4.5y | | 5y | Subacute encephalopathy after infection (dysarthria, dysphagia, disturbance of consciousness); seizures, status epilepticus; respiratory failure; developmental delay | | 4.5y | | focal seizures, generalized tonic-clonic seizures | | 1.midazolam, Esketamine 2.PB, LCM, CZP, OXC, LEV, VPA, TPM, PER 3. Ketogenic diet | | | 1.Mechanical ventilation; 2.Mitochondrial cocktail therapy | | |
| P8 | | M | 6mo | | 2y, 8mo | seizures | | 0.5y | | focal seizures | | 1.midazolam 2.OXC, VPA, LTG | | | 1.Mitochondrial cocktail therapy | | |
| P9 | | F | 6y | | 10y, 6mo | seizures, status epilepticus; dilated cardiomyopathy; respiratory failure | | 6y | | focal seizures, generalized tonic-clonic seizures, myoclonus | | 1.midazolam 2.LEV, VPA, PB, CLB, LTG, ZNS, PER | | | 1.Methylprednisolone 2.Mechanical ventilation 3.Mitochondrial cocktail therapy | | |
| P10 | | M | 3.5mo | | 8.5mo | seizures, developmental delay | | 3.5mo | | spasms, generalized tonic-clonic seizures, myoclonus | | LEV,TPM | | | 1.Mitochondrial cocktail therapy | | |
| P11 | | M | 7y, 5mo | | 7y, 11mo | seizures, status epilepticus, developmental delay, respiratory failure | | 7y, 5mo | | myoclonus | | PER, TPM, CLB | | | 1.Mitochondrial cocktail therapy | | |
| **ID** | **Outcome-mRS score** | | | **Plasma lactate** | | | **EEG** | | **Muscle/skin biopsy** | | **MRI** | | **Mutation** | **Domain** | **Inheritance** | **AD/AR** |  |
| P1 | 3 | | | Normal | | | Normal | | NA | | Acute phase:Slight thinning of corpus callosum | | c.445G>A, p.G149N | GTPase | De novo | AD |  |
| P2 | 5 | | | Normal | | | generalized epileptiform discharges, slowing background | | NA | | Acute phase:normal. Follow-up: cerebral atrophy | | c.1084G>A, p.G362S | Middle | De novo | AD |  |
| P3 | 4 | | | Normal | | | Slowing background and focal epileptiform discharges | | Basically normal | | Acute phase:normal. Follow-up: cerebral atrophy | | c.1207C>T, p.R403C | Middle | De novo | AD |  |
| P4 | - | | | Elevated | | | multifocal epileptiform discharges,RHADS | | NA | | Acute phase:normal. | | c.1207C>T, p.R403C | Middle | De novo | AD |  |
| P5 | 3 | | | Elevated | | | multifocal epileptiform discharges,RHADS | | NA | | Acute phase: edema of cortex. Follow-up: cerebral atrophy | | c.1207C>T, p.R403C | Middle | De novo | AD |  |
| P6 | 2 | | | Elevated | | | multifocal epileptiform discharges | | NA | | Acute phase: edema of right thalamus, and cortex;  Follow-up: cerebral atrophy | | c.1207C>T, p.R403C | Middle | De novo | AD |  |
| P7 | - | | | Normal | | | multifocal epileptiform discharges, slowing background | | NA | | NA | | c.1207C>T, p.R403C | Middle | De novo | AD |  |
| P8 | 2 | | | Elevated | | | focal epileptiform discharges | | NA | | Acute phase:normal. Follow-up: cerebral atrophy | | c.1085G>A, p.G362D | Middle | De novo | AD |  |
| P9 | 4 | | | NA | | | focal epileptiform discharges, slowing background | | NA | | Acute phase: edema of left cortex；Follow-up: cerebral atrophy | | c.1207C>T, p.R403C | Middle | De novo | AD |  |
| P10 | 3 | | | Normal | | | focal epileptiform discharges | | NA | | Acute phase:normal. Follow-up: cerebral atrophy | | c.1247T>C, p.L416P | Middle | De novo | AD |  |
| P11 | 4 | | | 1 | | | focal epileptiform discharges | | NA | | Acute phase: high signal of T2FLAIR in white matter.  Follow-up: cerebral atrophy | | c.1151A>C, p.H384P | Middle | De novo | AD |  |

LEV: Levetiracetam; VPA: Valproate; TPM: Topiramate; OXC: Oxcarbazepine; CZP: Clonazepam; LTG: Lamotrigine; LCM: Lacosamide; PER: Perampanel; CLB: Clobazam. NA: not available
