# Supplementary material for: Clinical and genetic characterization of DNM1l-related disorders: insights into genotype–phenotype correlations
Source: Front Pediatr. 2025 Oct 30;13:1672700. doi: 10.3389/fped.2025.1672700 (PMC12611810; doi:10.3389/fped.2025.1672700)
Supplement: Supplementary file 2 [file Table2.docx]

Table S2. Summary of key clinical features in patients with *DNM1L* variants in GED domain/AD and GTPase domain/AR groups.

| **Clinical data (n)** | **GED domain/AD**  **(n=3)** | **GTPase domain/AR (n=5)** |
| --- | --- | --- |
| **Median age of onset (years)** | 1.5 (range 0-3) | 0 (range 0-1) |
| **Died at the last follow-up** | 1 | 2 |
| **Development delay** | 2 | 5 |
| **Dystonia** | 2 | 4 |
| **Epilepsy** | 2 | - |
| **Status epilepticus** | 1 | - |
| **Ataxia** | - | 2 |
| **Microcephalus** | - | - |
| **Peripheral neuropathy** | 1 | - |
| **Failure to thrive** | - | - |
| **Cardiomyopathy** | - | - |
| **Optic atrophy** | 2 | - |
| **Abnormal brain MRI** | 1 | 3 |
| Cortical lesions | - | 1 |
| White matter lesions | - | 1 |
| Basal ganglia/brainstem lesions | - | 2 |
| Cerebral atrophy | 1 | - |
| Corpus callosum thinning/absence | - | - |
| **Lactate peak in MRS** | - | 2 |
| **Abnormal EEG** | 1 | - |
| Epileptiform discharge | - | - |
| Slow background | 1 | - |
| RHADS | - | - |
| **Hyperlactacidemia** | - | 2 |
| **Abnormal in muscle biopsy** | - | 2 |
| Abnormal morphology of mitochondria | - | - |
| Decreased respiratory chain enzyme activity | - | 2 |
| Nonspecific findings | - | - |
